# Annotations

**Published:** 1886-12-18

**Authors:** 


### ANNOTATIONS.

The Hospitals Week Movement.

The success which attended the new departure made by the Council of the Metropolitan Hospital Sunday Fund in June last, by which a series of meetings were held throughout the metropolis on each of the six days preceding Hospital Sunday, has been recognised by the provincial towns. Leeds, one of the most active centres of provincial life, has adopted the movement with marked success. A series of seven public meetings has been held in Leeds in support of the movement for increasing the work-people's subscriptions to the medical charities in that town. An endeavour is being made to organise regular weekly or fortnightly subscriptions in workshops and factories throughout the district on behalf of all the medical charities. With this view, the meetings were arranged to take place in each of seven districts of the town, and in every instance several working men came forward and joined the ward committee at the close of the proceedings. It has been decided that the work-people's subscriptions shall be paid into one general fund, and afterwards divided amongst the charities in proportion to the amounts which each received from work-people at the end of the last financial year. The inducements offered to the work-people were: the admission of their representatives as governors of the various charities, and a voice in their management. It is hoped that £8,000 per annum will be raised by work-people's subscriptions through the Hospitals' Week movement, the whole amount now received being about £2,000 per annum. We wish the movement all success, and commend the example of Leeds to hospital managers throughout the country.

Temperance and Total Abstinence.

The Archbishop of York, speaking at a meeting at the Temperance Hall, Middlesborough, a few days ago, said "that the Church of England Temperance Society had a double basis; it admitted those who were temperate and those who were total abstainers. He had seen the almost total shipwreck of a temperance mission in a well-known place, owing to a determined onslaught on a gentleman who said he preferred being a temperance man to being a total abstainer." Every person familiar with the medical aspects of the alcohol question cannot but sympathise deeply with the temperance movement. It is quite certain that if alocholic drinks were banished from the face of the earth it would be an almost unmixed good. At the same time it cannot be denied that to some persons a small quantity of alcohol is certainly not injurious, whilst to others it is almost as certainly a positive advantage. In any case, it is unreasonable and unchristian to make total abstinence a test of morals or religion. To commence an onslaught upon a person because he declares for temperance rather than total abstinence is the very madness of fanaticism. The middle course is proverbially the safest, and in such a matter as the abstaining or not from alcoholic drinks, where expediency and not principle is involved, one would think that a large majority would prefer the middle course. That however is not always so, and it not infrequently happens that the intemperance in speech of those who abstain from intoxicating drinks does more injury to the cause of true temperance than their total abstinence from alcohol can counterbalance.

A Hospital for Oban.

The town of Oban in Argyleshire, is perhaps the best-known resort which the West of Scotland can boast, and now that the thousands of tourists and pleasure-seekers who annually visit it, have left and returned to their varied avocations, it is waking up to a perception of its own immediate needs. The place is the head-quarters of the fishing industry on the north-west coast; and to Oban are brought the cases of accident which not infrequently occur amongst those that "go down to the sea in ships, and occupy their business in great waters." Such being the case, it might naturally be supposed that the inhabitants have long since provided themselves with some special means for the treatment of their own sick, and of the sufferers who are brought to their doors. But it is not so. The only local institution for the aid of those suffering from accident or disease, is the Lorn Combination Poorhouse, and no one with any knowledge or experience of such matters will venture to declare that this accommodation is likely to be in any way adequate for the needs of the district. Those who cannot be admitted to, or are unwilling to enter the doors of, the Poorhouse, are compelled, if their own homes have no suitable provision, to seek admission to one of the large infirmaries in the south, say at Edinburgh or Glasgow. The long journey which such a course entails cannot but aggravate the symptoms of the unfortunate invalid who is obliged, perhaps in the depth of such a winter as Scotland often knows, to undertake it. Now, if some one holding high official position, as, for instance, the Provost of Oban, were to come forward and declare how great is the public need, we feel sure that his endeavours would be ably seconded, not only by those who, living in and around Oban, cannot fail to be cognisant of the facts here set forth; but also by the numerous English visitors who, in the summer time, seek health and recreation at Oban; and by the owners of the great fleet of yachts which annually congregate in the waters of this well-known bay, at the head of the Firth of Lorn. A word to the wise is sufficient.

---

A young and handsome doctor was advising a lady patient with respect to her health. "You must really take more exercise, I assure you," urged the doctor. "I'll do that with pleasure," rejoined the interesting patient; "I'll jump at the first offer." They are married now.

